# Twists and turns: CRT-D with mixed Twiddler and Reel syndromes

**DOI:** 10.1007/s12471-024-01917-0

**Published:** 2024-12-18

**Authors:** Pierre Rossignon, Riad Tajildin, Edith Famdie

**Affiliations:** 1 Department of Cardiology, Centre Hospitalier de Luxembourg, Luxembourg City, Luxembourg; 2 Department of Cardiology, CHU Sart Tilman, Liège, Belgium

**Fig. 1:**
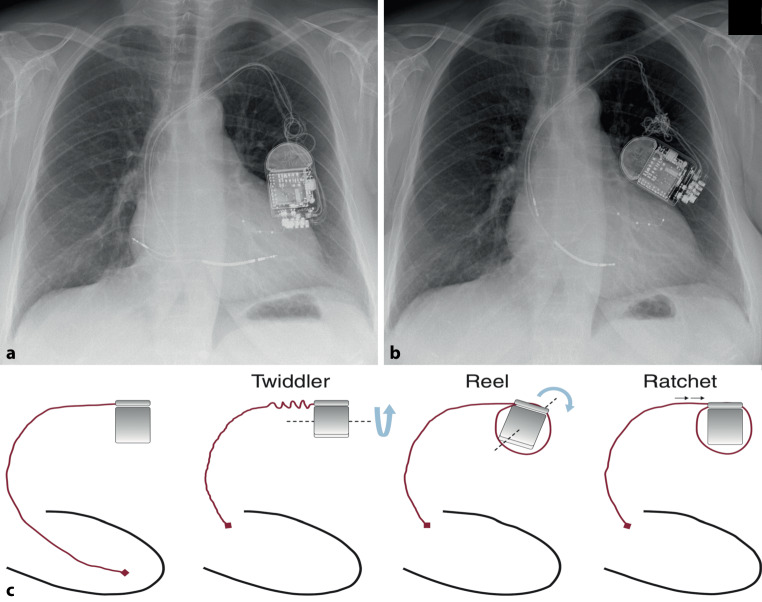
**a** Chest radiography taken four months earlier for an unrelated purpose, already showing a rotated generator with some lead twisting. However, it was not reviewed by a cardiologist at that time. **b** Chest radiography at presentation, revealing both Twiddler and Reel syndromes, with leftward rotation of the pulse generator, CRT‑D lead twisting, and dislodged atrial and right ventricular leads. The left ventricular lead remained properly positioned. The intense twisting and progression between (**a**) and (**b**) suggest compulsive generator manipulation by the patient. **c** Movements involving the generator or leads contributing to various macro-dislodgement syndromes. Reproduced with permission from Elsevier [1] ©. Not covered by this article’s CC BY 4.0 License. Reuse requires Elsevier’s permission

A 65-year-old woman with a cardiac resynchronisation therapy-defibrillator underwent routine device interrogation, revealing abnormalities in the atrial lead (reduced amplitude and occasional detection failure) and the right ventricular lead (increased pacing threshold) 13 months post-implantation. This prompted a chest X‑ray due to suspected lead dislodgement (Fig. 1b).

The X‑ray revealed twisted and macro-dislodged leads, along with pulse generator rotation, indicative of both Twiddler and Reel syndromes. These syndromes result from specific mechanisms of macro-dislodgement caused by movements of the generator or leads ([1]; Fig. 1c).

Potential risk factors are debated and include a psychiatric history, obesity, advanced age, an oversized pocket, manual manipulation, and the absence of generator fixation [2]. In our patient, the last two factors were present. Suturing the generator may be the most effective preventive strategy [3], although it is considered optional [4].

Even in the presence of risk factors, there are no specific guidelines recommending increased follow-up, reimplantation procedures, or routine radiography. However, routine imaging can be useful in identifying dislodgement [5] and is therefore advisable in conjunction with routine electrical testing. Lead revision is definitively recommended in cases of lead dysfunction [4].
